# Healthcare utilization among aging Latvians with diminished activities of daily living

**DOI:** 10.1093/eurpub/ckac131.579

**Published:** 2022-10-25

**Authors:** C Queen, R Pasupathy, I Reine

**Affiliations:** 1 Department of Public Health, Texas Tech University Health Sciences Center, Abilene, USA; 2 Institute of Public Health, Riga Stradins University, Riga, Latvia; 3 Department of Public Health and Caring Sciences, Uppsala University, Uppsala, Sweden

## Abstract

The population in Latvia is aging. Independence in performing activities of daily living (ADL) is a core aspect of functioning, and the elderly frequently experience limitations in functioning. Little is known about the utilization of healthcare of elderly Latvians with functional difficulties. The purpose of this study was to determine the relationship between functional difficulties and utilization of healthcare among the elderly in Latvia. This study had three overall objectives: (i) to investigate the determinants of utilization of health care for elderly Latvians with functional difficulties; (ii) examine the relationship between predisposing characteristics, enabling resources, and need with specific measures of access to care using the Behavioral Model for Vulnerable Populations and to (iii) identify the nature and existence of health disparities among the elderly in Latvia, with and without functional difficulties. Data from the 2017 Survey of Health, Ageing and Retirement in Europe (SHARE) survey, with a sample size of 1479 was utilized. There was a statistically significant difference in the utilization of healthcare between individuals with and without functional disabilities (F(4,1) =759.615, p < 0.01), with a higher utilization of healthcare among individuals with functional difficulties (p< .01). The results of Automatic Linear Modeling indicate that significant (p < 0.05) factors in predicting utilization of healthcare include factors such as age, public sickness benefit and disability pension, and overall health status. This study is significant because it fills critical gaps in knowledge that exist with respect to healthcare utilization for elderly Latvians with functional disabilities.

**Key messages:**

• This study contributes to our understanding of healthcare utilization of aging Latvians.

• This study provides insight into the functional limitations of the aging Latvian population.

